# EBFA-6D: End-to-End Transparent Object 6D Pose Estimation Based on a Boundary Feature Augmented Mechanism

**DOI:** 10.3390/s24237584

**Published:** 2024-11-27

**Authors:** Xinbei Jiang, Zichen Zhu, Tianhan Gao, Nan Guo

**Affiliations:** 1 Software College, Northeastern University, Shenyang 110004, China; elasunaming@stumail.neu.edu.cn (X.J.); zhuzichen@stumail.neu.edu.cn (Z.Z.); 2 School of Computer Science and Engineering, Northeastern University, Shenyang 110004, China; guonan@mail.neu.edu.cn

**Keywords:** 6D pose estimation, deep learning, transparent objects, attention mechanism

## Abstract

Transparent objects, commonly encountered in everyday environments, present significant challenges for 6D pose estimation due to their unique optical properties. The lack of inherent texture and color complicates traditional vision methods, while the transparency prevents depth sensors from accurately capturing geometric details. We propose EBFA-6D, a novel end-to-end 6D pose estimation framework that directly predicts the 6D poses of transparent objects from a single RGB image. To overcome the challenges introduced by transparency, we leverage the high contrast at object boundaries inherent to transparent objects by proposing a boundary feature augmented mechanism. We further conduct a bottom-up feature fusion to enhance the location capability of EBFA-6D. EBFA-6D is evaluated on the ClearPose dataset, outperforming the existing methods in accuracy while achieving an inference speed near real-time. The results demonstrate that EBFA-6D provides an efficient and effective solution for accurate 6D pose estimation of transparent objects.

## 1. Introduction

Estimating the 6-DoF pose, i.e., the 3D translation and 3D rotation, of objects is a fundamental problem in computer vision with broad applications, including autonomous driving, robotics, and augmented reality technologies [1,2,3]. Recent years have witnessed tremendous progress in this area, with current methods [4,5,6] achieving high accuracy on datasets featuring opaque objects [7,8,9,10,11]. However, transparent objects, which are frequently encountered in everyday environments, have received limited attention. The challenges arise from two primary factors. First, transparent objects lack inherent texture or color, which undermines the visual cues to distinguish them from their background. Second, most commercial sensors based on LiDAR or stereo vision struggle with transparent surfaces, as transmitted light passes through the object or refracts within the object, resulting in incorrect depth information. As shown in [12], even state-of-the-art methods show a significant performance gap when applied to transparent objects, despite being trained and tested with ground truth depth data.

To address these challenges, previous studies either leveraged multi-view information to reconstruct scene geometry or trained a depth completion network to enhance the generalization of RGB-D-based methods to transparent objects. While multi-view approaches [13,14] show robustness in handling occlusion, they require special hardware setups (e.g., multiple cameras to capture different perspectives), which limits their practicality in real-world applications. On the other hand, depth completion approaches [15,16,17,18] can recover object poses from a single image, but the additional computational overhead introduced by these networks makes real-time pose estimation challenging, particularly for resource-constrained systems.

Recently, RGB-based methods [19,20] have emerged, unlocking new applications such as augmented reality on mobile devices. These methods typically use a standard object detector to predict regions of interest then predict dense correspondences for each object and solve object poses using PnP and its variants. Despite producing promising results, these methods suffer significant slowdowns as the number of objects presented in the image increases, making them less suitable for real-time applications with multiple objects.

**Figure 1:**
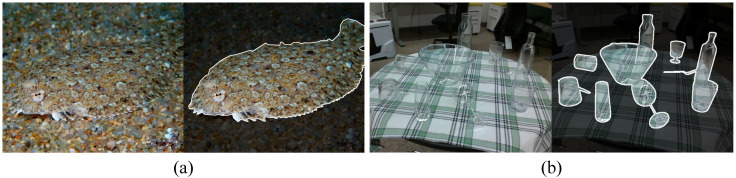
Illustration of the camouflaged object detection task (**a**) and transparent object detection task (**b**). Camouflaged and transparent objects share similar internal textures with their surroundings, making them challenging to detect. However, they exhibit relatively distinct boundaries that can be leveraged for detection.

In this work, we propose EBFA-6D, a novel end-to-end RGB-based method for transparent object pose estimation. Unlike previous RGB-based approaches, our method formulates pose estimation as a multi-task learning problem. The model simultaneously predicts the object’s bounding box and pose directly from a single image. To improve generalization to transparent objects, as shown in Figure 1, we draw inspiration from camouflaged object detection and leverage boundary information as a consistent visual clue to enhance the performance for transparent object perception. To adapt the boundary guidance from semantic segmentation for camouflaged objects to pose estimation task for transparent objects, we introduce a novel Boundary Feature Augmented Mechanism (BFAM). In detail, we designed a simple yet effective Transparency-Aware Boundary Prediction Module (TBPM) to predict boundaries and incorporate a Boundary Focus Module (BFM) to help the model focus on boundary information. Meanwhile, we introduce a Bottom-up Feature Fusion Module (BFFM) to further enhance the capability of localizing the object in EBFA-6D.

Our main contributions are summarized as follows:

1. We propose EBFA-6D, a novel end-to-end pose estimation framework for transparent object pose estimation. By leveraging boundary cues, our approach significantly improves the accuracy and robustness of transparent object pose estimation while achieving near real-time performance.
2. We introduce the Boundary Feature Augmented Mechanism (BFAM), which consists of the Transparency-Aware Boundary Prediction Module (TBPM) and the Boundary Focus Module (BFM) to enhance boundary feature extraction with minimal computational overhead, thereby focusing the network more effectively on key boundary features.
3. Our experiments demonstrate that EBFA-6D achieves state-of-the-art performance on ClearPose dataset, demonstrating the effectiveness of our method.

The remainder of this paper is organized as follows. In Section 2, we briefly review the related work in 6D pose estimation for transparent objects. The proposed method is introduced in Section 3, and we present the experimental results in Section 4. Finally, we conclude the paper in Section 5.

## 2. Related Work

Over recent decades, 6D pose estimation has been extensively studied, with numerous methods achieving high accuracy on datasets of diffuse objects. These methods can be mainly classified into two categories. The first category of methods [21,22,23] directly predict the 6D pose; the second category [5,24,25,26,27] establishes a mapping between 2D and 3D, followed by RANSAC-PnP solvers to estimate the pose. However, most of these methods focus on solving the pose estimation problem of textureless or occluded objects with opaque surfaces, with few studies addressing transparent objects. The unique visual properties of transparent objects, such as light refraction and minimal texture cues, introduce significant challenges to these conventional methods. In this section, we first revisit recent advancements in 6D pose estimation for transparent objects, focusing on datasets and methods in Section 2.1. We then discuss related work on regression-based pose estimation methods in Section 2.2, which share similarities with the proposed approach.

### 2.1. 6D Pose Estimation for Transparent Objects

Due to the scarcity of 6D pose datasets for transparent objects, significant research efforts have been devoted to bridging this gap. One notable contribution is ClearGrasp [15] proposed by Sajjan et al., which addresses the challenge of data acquisition for transparent objects by leveraging synthetic data. ClearGrasp provides an RGB-D dataset comprising over 50,000 synthetic images, as well as 286 real-world images used for evaluation. Similarly, Fang et al. introduced TransCG [17], a dataset consisting of 57,715 real RGB-D images collected from 130 different desktop setups featuring various transparent objects. However, the ground truth poses in TransCG are obtained by localizing markers attached to the objects, which are inconsistent with real-world application scenarios. To bridge the gap between synthetic and real-world data, Ikeda et al. [28] applied domain adaptation techniques to enhance the accuracy of transparent object pose estimation. Inspired by prior studies [29,30,31], Ikeda et al. established a weak pairwise correlation between synthetic and real-world data and trained an instance-level style transfer network to render target objects in synthetic images with a more realistic appearance. However, due to the sensitivity of transparent objects to environmental influences, this method struggles to accurately simulate physical textures in complex object placement scenarios. Chen et al. introduced ClearPose [12], a large-scale real-world dataset for transparent object estimation, along with a semi-automatic annotation tool, ProgressLabeller [32]. ClearPose comprises over 350,000 real-world RGB-D images of transparent objects, captured across diverse scenarios without the use of localization markers, ensuring the dataset remains unbiased. This dataset reflects the complexity and variability encountered in real-world environments, making it a valuable resource for advancing research in transparent object pose estimation. Given its comprehensiveness and real-world relevance, we selected ClearPose for the experiments conducted in this study.

Previous research [33,34] has attempted to use hand-crafted features for pose estimation in transparent objects. [33] proposed to segment objects based on invalid depth value, followed by edge-based geometric hashing for initial pose estimation, and subsequently refines the pose through Iterative Closest Point (ICP) algorithm. Similarly, [34] first segmented transparent candidates by identifying depth discontinuities and then used SIFT features to detect transparency by analyzing background distortion. Their method combines stereo matching of RGB and infrared (IR) contours to reconstruct 3D object models, facilitating robotic grasping tasks. Although these methods have contributed to the field, they exhibit several limitations. Being predominantly rule-based, they depend heavily on geometric and feature-engineered techniques, making them reliant on specific sensor modalities and predefined heuristics. Consequently, their generalization to diverse environments is limited, particularly in scenarios with complex occlusions.

In contrast, deep learning has demonstrated its capability to learn intricate features from data [35], which provided more robust solutions for pose estimation for transparent objects. As the transparency property fails sensors to capture precise depth data, RGB-D-based methods for general objects struggle to generalize to transparent objects. Recent methods have taken two primary directions to address this challenge: refining depth data through depth completion netoworks or directly estimating poses using RGB data.

For methods leveraging depth information, Li et al. [18] proposed FDCT, a fast depth completion method based on raw RGB-D data. This method optimizes low-level features by incorporating fusion branches, feature fusion and extraction modules, and improving the loss function. However, it also leads to a dependence on low-level features, which is not robust enough against sensor noise. Initially designed for natural language processing (NLP), the Transformer architecture has proven capable of modeling global relationships and scale efficiently with model size and training data [36], making it adaptable to a variety of tasks. Dai et al. [37] introduced SwinDRNet, a depth completion network based on the dual-stream Swin Transformer [38]. The network first extracts multi-scale features from RGB and depth maps, using cross-attention for feature fusion. They trained their network with the synthetic RGB-D dataset DREDS, which simulates real sensor noise, and evaluated it on the real-world dataset STD. Xu et al. [39] proposed a two-stage pose estimation method. In the first stage, surface normal recovery and RANSAC plane estimation are applied after segmentation. In the second stage, an extended point-cloud representation consisting of normals, plane depths, and UV coordinates are used for pose estimation. Zhang et al. [40] proposed a two-stage category-level pose estimation network called TransNet. The first stage segments the object and extracts depth and surface normal information, while the second stage uses Transformer [41] for object pose and scale prediction. However, the methods above [37,39,40] involve multiple processing modules and are not end-to-end, which results in high computational overhead and limits generalizability.

Among RGB-based methods, Weidenbach et al. [20] improved pose estimation by replacing the modal annotations for masks and bounding boxes to amodal annotations, without modifying the underlying network architecture. Yu et al. [19] introduced TGF-Net, a model leveraging geometric feature fusion, and introduced the synthetic dataset Trans6D-32K, demonstrating TGF-Net’s capability for domain adaptation and robustness. However, these methods require training a separate network for each of the *N* object instances, resulting in an inference complexity of O(N), restricting their usability for scenarios requiring simultaneous recognition of multiple objects.

In summary, existing RGB-D-based methods for transparent object pose estimation heavily rely on depth completion networks to capture geometry details. While RGB-based methods alleviate the need for depth data, they still have a noticeable performance gap. Both approaches face significant challenges, including high computational costs from multi-stage pipelines and a lack of end-to-end efficiency, which hinder their real-time applicability. To address these limitations, we propose an approach to directly recover object poses from RGB data, aiming for both efficiency and robustness.

### 2.2. Regression-Based Pose Estimation Methods

Direct regression methods, which convert 6D pose estimation into regression and classification tasks, have emerged as a promising approach to reduce algorithmic complexity while maintaining high performance. One of the foundational works is PoseCNN [10] proposed by Xiang et al. PoseCNN is an end-to-end network that regresses object pose parameters from a single RGB image. Notably, they decoupled 6D pose estimation into two tasks: 3D translation and 3D rotation. For translation, PoseCNN employs a Hough voting mechanism, where the network predicts pixel-wise unit vector pointing towards the object’s center to handle occlusions. To handle rotational uncertainty introduced by symmetric objects, PoseCNN adopts the ShapeMatch-Loss, leveraging object geometry to overcome ambiguity. Kehl et al. [21] proposed SSD-6D, which builds upon the object detection framework SSD [42]. SSD-6D transforms rotational estimation into a classification problem by discretizing the rotation space into discrete bins. This discretization simplifies the rotation estimation process and allows for efficient inference. However, it introduces quantization errors and limits rotational precision compared to continuous regression methods. Selvam et al. introduced YOLOPose V2 [43], which replaced the conventional CNN architecture with Transformer [41] architecture for single-stage, end-to-end object detection and pose estimation. The multi-head self-attention mechanism in Transformer captures global dependencies in the feature maps, enhancing the global feature representation and improves the performance. However, Transformers typically require large amounts of data to generalize effectively. In scenarios with limited training data, their performance gains may diminish, and they may exhibit slower inference times compared to CNN-based methods. COPE [44] proposes an end-to-end network architecture that utilizes geometric correspondences as intermediate representation to directly estimate 6D object pose. Built upon Ref. [45], this approach not only makes the network end-to-end trainable but also enables constant-time inference. Consequently, it is applicable and useful for a broad variety of real-time tasks.

In conclusion, for regression-based methods, numerous studies typically build upon existing object detection architectures, offering a balance between speed and accuracy. Due to the lightweight nature and convenience of single-stage object detection frameworks, along with their inherent capability for multi-object recognition, these studies additionally design subnetwork to adapt existing detection framework to object pose estimation task. However, current methods still struggle with complex scenes, occlusions, and symmetric objects. Our proposed approach follows this line of work, specifically, using COPE’s geometric correspondence supervision to accelerate inference and improve practicality for transparent object pose estimation.

## 3. The Proposed Method

Given an input RGB image *I* and a set of N objects and their CAD models $M=\{M_{1},M_{2},\ldots ,M_{N}\}$, our goal is to predict all the objects’ transformations [R|T], with 3D rotation R∈SO(3) and 3D translation $T\in R^{3}$ with respect to camera coordinate system.

**Figure 2:**
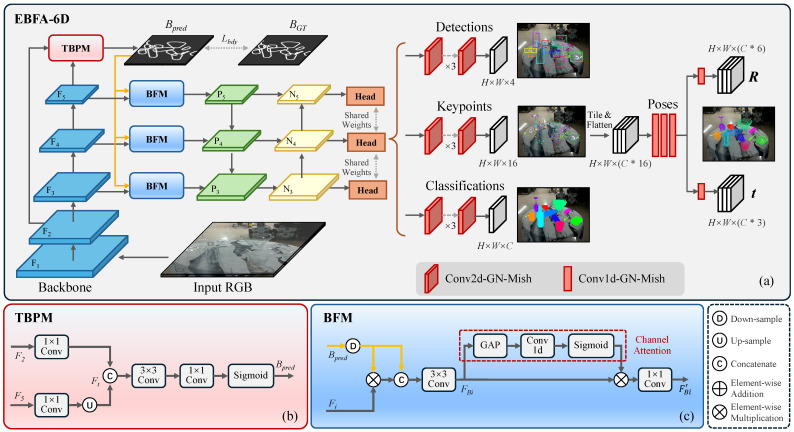
The network architecture of EBFA-6D. (**a**) Overall architecture showing the feature extraction backbone and key modules. (**b**) Detailed structure of TBPM (Transparency-Aware Boundary Prediction Module), used to predict boundary maps. (**c**) The structure of BFM (Boundary Focus Module), which utilizes channel attention to enhance features.

The architecture of the proposed EBFA-6D is illustrated in Figure 2. Our approach formulates instance-level pose estimation as an end-to-end learning task based on FCOS [45], using a multi-task learning strategy (Section 3.1). To improve the model’s ability to detect transparent objects, we propose Boundary Feature Augmented Mechanism (BFAM), where we propose the novel transparent-aware boundary, and design the Transparency-Aware Boundary Prediction Module (TBPM) and Boundary Focus Module (BFM) (Section 3.2). To better preserve fine spatial details while integrating high-level semantic information, we introduce the Bottom-up Feature Fusion Module (BFFM)to further enhance the feature quality (Section 3.3). Finally, we detail the design of loss functions and the post processing strategy employed to filter redundant estimations (Section 3.4 and Section 3.5).

### 3.1. End-to-End Multi-Instance Pose Estimation Framework

Previous RGB-based methods [19,20] for transparent objects typically treat the pose estimation process as a two-stage task. In the first stage, a standard object detector extracts the object bounding boxes, and the foreground region of the image is cropped and resized to a fixed shape. In the second stage, each cropped image is passed through another network to segment the object and predict dense 2D–3D correspondences. These correspondences are then utilized by Perspective-n-Point (PnP) algorithms to recover the object’s pose. However, these approaches require repeating the second stage for each object, leading to inefficient performance, especially in crowded scenes.

To this end, we designed our pose estimation network in a one-stage manner, e.g., performing both detection and pose estimation at the same time. This enables the network to estimate the poses of multiple objects and instances with a single forward pass. To bridge the gap between detecting objects and estimating poses in different dimensional spaces, we introduce an intermediate task of detecting the projected keypoints at the corners of the object’s bounding boxes.

The structure of EBFA-6D consists of three main components: the feature extraction component, the feature fusion component, and the prediction component. The feature extraction component is responsible for extracting features from the input image, utilizing the ResNet-101 network. As shown in Figure 2a, the output feature layers of the network backbone are denoted as $F_{1}$ to $F_{5}$.

The extracted features $F_{3},F_{4}$, and $F_{5}$ from the backbone network are fed into the feature fusion module that utilizes a feature pyramid network (FPN) structure to integrate high-level semantic information with low-level data, producing a more comprehensive feature representation. The output feature maps $P_{3},P_{4}$, and $P_{5}$ retain resolutions consistent with their corresponding input layers. For instance, an input image of size 640×480 undergoes five stages of downsampling, resulting in $P_{3},P_{4}$, and $P_{5}$ with resolutions of 80×60,40×30, and 20×15, each corresponding to different target sizes.

The prediction module is built on a fully convolutional network structure that performs pixel-wise prediction and shares weights across feature maps at different levels. It consists of three main branches: classification, bounding box regression, and keypoint prediction branches. Each branch is composed of three layers of 2D convolution and GroupNorm, with Mish as the activation function for the hidden layers. For each of the *N* locations on the feature map, the classification branch predicts the class probability vector $\hat{C}=\left\{{\hat{c}}_{1},{\hat{c}}_{2},...,{\hat{c}}_{N}\right\}$, and the bounding box regression branch predicts $\hat{B}=\left\{{\hat{b}}_{1},{\hat{b}}_{2},...,{\hat{b}}_{N}\right\}$, where ${\hat{b}}_{N}\in R^{4}$. The keypoint regression branch predicts $\hat{K}=\left\{{\hat{k}}_{1},{\hat{k}}_{2},...,{\hat{k}}_{N}\right\}$, where ${\hat{k}}_{N}\in R^{16}$ represents the 2D positions of the projections of the eight corner points of the object’s bounding box in the image. Based on the predicted keypoints, the pose prediction branch predicts the object’s pose $\hat{P}=\left\{{\hat{p}}_{1},{\hat{p}}_{2},...,{\hat{p}}_{N}\right\}$, where ${\hat{p}}_{N}\in R^{9}$. Three parameters represent the object’s 3D translations $\hat{T}$, and six parameters represent the 3D rotations $\hat{R}$, as proposed by [46]. Both $\hat{R}$ and $\hat{T}$ are predicted using three 1D convolutional layers.

### 3.2. Boundary Feature Augmented Mechanism

Transparent objects, which lack inherent texture attributes, are highly sensitive to changes in the background, viewing angle, and environmental lighting. However, the boundaries of these objects consistently exhibit the highest contrast, making it a reliable feature for guiding the network in localizing transparent objects. This makes boundary information more effective than texture for network processing in pose estimation tasks. Despite these challenges, the boundaries of transparent objects often provide distinct and stable high-contrast features, making them an effective visual cue for guiding the network in localizing such objects. Consequently, utilizing boundary information proves more effective than relying solely on texture cues for pose estimation tasks involving transparent objects.

To this end, we propose to integrate boundary prediction as an auxiliary task into the pose estimation framework, leveraging the boundary’s stability and high contrast inherent to transparent objects. Specifically, we introduce TBPM to predict the object boundaries. Subsequently, BFM is employed to guide the network’s focus on boundary information using an attention mechanism, offering a robust and complementary feature source for improved localization and pose estimation.

#### 3.2.1. Transparency-Aware Boundary Prediction Module

The challenge of transparent object pose estimation shares similarities with camouflaged object detection, as both tasks face difficulty relying on internal texture for object localization. To address this, we draw inspiration from previous camouflaged object detection methods [47,48], incorporating a boundary prediction branch to enhance object localization in images.

**Figure 3:**
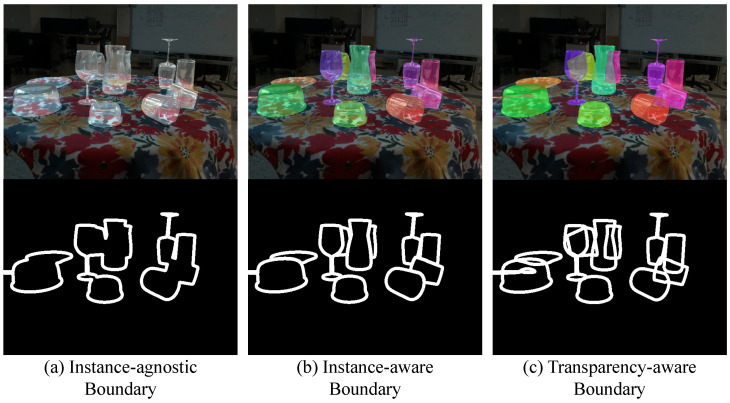
Three different pixel-level supervision methods. (**a**) Instance-agnostic Boundary denotes the boundary pixels of the region common to all objects in each image. (**b**) Instance-aware Boundary denotes the boundary pixels of each object within the camera view. (**c**) Transparency-aware Boundary denotes the boundary pixels of each object after its boundary have been superimposed.

While camouflaged object detection primarily requires pixel-level distinction between foreground and background in an instance-agnostic way, illustrated in Figure 3a, our task is more complex, as it involves predicting the 6D pose of each object instance. To address this challenge, we propose a novel Transparency-Aware Boundary representation, as shown in Figure 3c, which captures the complete boundary of objects, even when occluded. Our key insight is that transparent objects remain distinguishable, even when partially occluded by others. We also compare three different pixel-level supervision methods, as shown in Figure 3 in Section 4.4.

To facilitate efficient boundary prediction without significant computational overhead, we designed a simple yet effective boundary prediction subnetwork, TBPM. As shown in Figure 2b, TBPM takes the backbone’s features as input and predicts the complete boundary of the object in the image. While the initial stages of the network can extract a broad range of boundary information, these boundaries extracted may not always correspond to the object of interest. To help the network focus on the relevant object, it is crucial to integrate low-level features, which capture local boundaries, with high-level semantic features that provide global context. To achieve this, we select the low-level feature map $F_{2}$ and the high-level feature map $F_{5}$. First, the channels of $F_{2}$ and $F_{5}$ are mapped to 64 and 256 dimensions, respectively, using 1×1 convolutional layers. Then, the high-level features are upsampled using bilinear interpolation to match the resolution of the low-level features. The two feature maps are then concatenated to produce the feature map $F_{t}$. Finally, the single-channel boundary feature map $B_{pred}$ is obtained by applying the sigmoid function after convolving $F_{t}$ with 3×3 and 1×1 kernels.

#### 3.2.2. Boundary Focus Module

The transparent objects often result in refraction and reflection, which introduce ambiguity in the interpretation of boundaries. To integrate boundary information in the feature map, we propose the Boundary Focus Module (BFM), inspired by SENet [49]. This module enhances boundary perception by focusing on transparent object features. By jointly training boundary extraction and pose estimation models, BFM allows the network to integrate both global and local features, improving its ability to discern the boundaries of transparent objects.

As shown in Figure 2c, BFM combines the feature maps $F_{3},F_{4}$, and $F_{5}$, with the predicted boundary map $B_{pred}$ generated from TBPM. $B_{pred}$ is downsampled to match the feature map $F_{i}$, where i∈[3,5], followed by an element-wise multiplication operation. The resulting feature maps are concatenated with $B_{pred}$ and passed through a 3×3 convolutional layer to extract the deeper boundary-aware features, denoted as $F_{Bi}$.

In the subsequent step, the feature map $F_{Bi}$ undergoes global average pooling, where all channel features are encoded into a single global feature. This global feature is then used to capture the nonlinear interconnections between channels through an activation weighting operation. To achieve this, two fully connected layers are used to learn the channel-wise weights, which are then compressed into the range [0,1] using a sigmoid function. These weights are applied channel-by-channel to $F_{Bi}$ resulting in the boundary feature map $F_{Bi}^{\prime }$, which is weighted by the boundary information. With BFM, the model can effectively focus on the distinct boundaries of transparent objects, thereby improving performance in complex scenes.

### 3.3. Bottom-Up Feature Fusion Module

In modern object detectors, it is common practice to employ FPN to extract multi-scale features. The FPN enhances small object detection by propagating semantic information from higher levels to lower levels. However, since our approach leverages low-level boundary features to improve transparent object perception, it is essential to preserve the rich details from lower-level features. To achieve this, we introduce a Bottom-up Feature Fusion Module (BFFM) following the feature pyramid module.

The bottom feature map $P_{3}$ from the feature pyramid module is used as the bottom feature map $N_{3}$ in the feature fusion module. A 3×3 convolution with a stride of 2 is applied to $N_{3}$ and produce $N_{3}^{\prime }$, ensuring that $N_{3}^{\prime }$ has the same size as the feature map $P_{4}$. Then, $N_{3}^{\prime }$ and $P_{4}$ are concatenated to obtain $N_{4}$. Similarly, the module outputs the fused feature map $N_{5}$. As a result, information from the feature map $F_{3}$ traverses fewer layers to reach the top layer feature map $N_{5}$. In this way, we enhance the model’s localization ability by facilitating the flow of low-level information to the high-level feature map through multi-scale information aggregation.

**Figure 4:**
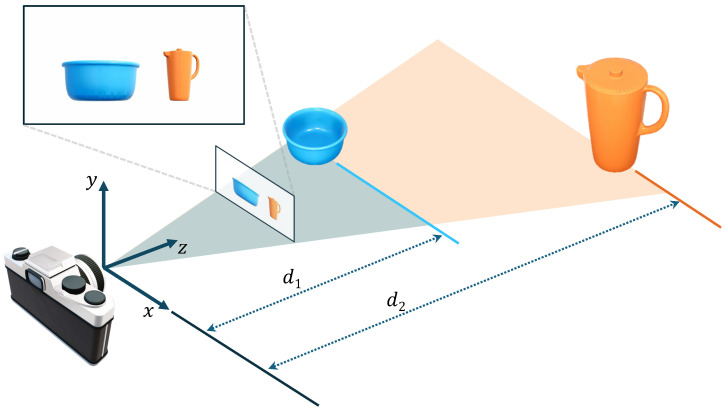
Illustration of the effect of object distance on perceived size in monocular vision. Although the orange pitcher is physically larger than the blue bowl, the blue bowl appears larger from the camera’s perspective due to its closer proximity.

To determine the level of pyramids responsible for predicting the objects, the standard approach is to assign objects to specific feature pyramid levels based on their bounding box size. However, as shown in Figure 4, when depth information is uncertain in RGB data, the network may inaccurately estimate the object scale. Given that our primary objective is to estimate the pose of objects in 3D space, it is essential to address the issue of depth ambiguity problem and incorporate scale information of objects during training. To this end, we optimize the strategy for assigning objects to different pyramid levels.

During training, we preprocess the input data by calculating the ratio of the target 3D model’s size to its distance from the camera. Since the features extracted from input image are exponentially downsampled, a logarithmic function is used to determine the most appropriate feature layer. Let the 3D model be denoted as *M*, with $V_{M}$ representing the set of all mesh vertices on *M*, where $v_{i},v_{j}\in V_{M}$, and i≠j. The level which the object should be assigned can be computed using Equation (1):

(1) $$level=n+log_{\sigma }\left(\frac{max||v_{i}-v_{j}{\left|\right|}_{2}}{t_{z}}\right)$$

where level∈[3,5], corresponding to the feature maps $N_{3}$ to $N_{5}$ produced by the feature fusion module. *n* represents the layer of feature maps, and in our experiment, we set n=4, using $N_{4}$ as the basis for selection, $t_{z}$ denotes the distance between the center of the target 3D model and the camera along the z-axis, and σ is a hyperparameter, which is set to σ=3 here.

### 3.4. Loss Function

EBFA-6D is a pose estimation method that extends the object detection framework by adding keypoint prediction branches to the original detection task, allowing it to predict object poses based on keypoints. To help the model focus on boundary information, which is crucial for transparent object identification, we have incorporated a boundary prediction branch. As a result, EBFA-6D can be considered a multi-task learning model, with the loss function for each task shown in Equation (2):

(2) $$L_{total}=L_{det}+L_{bdy}+L_{pe}.$$

Given the limitations of commonly used loss functions like mean absolute error (MAE) and mean square error (MSE) in handling outliers, the regression tasks in EBFA-6D are computed using huber loss. The huber loss HB(a,b) combines the strengths of MAE and MSE, as shown in Equation (3):

(3) $$HB\left(a,b\right)=\left\{\begin{array}{c} \frac{1}{2}{\left(a-b\right)}^{2},\left|a-b\right|\leq \delta ,\\ \delta |a-b|-\frac{1}{2}\delta^{2},otherwise. \end{array}\right.$$

where *a* represents the ground truth and *b* represents the predicted value, while the hyperparameter δ controls the balance between MAE and MSE in the Huber loss. By combining the advantages of both MAE and MSE, huber loss reduces sensitivity to outliers and improves the overall model performance.

In transparent object pose estimation, we account for the imbalance between foreground and background, as well as the disproportionate number of examples across categories due to varying object sizes. To address this, we adopt Focal Loss [50], which aids in learning from difficult examples and improves the accuracy of detecting small objects. The expression for Focal Loss is shown in Equation (4), where $p_{t}$ represents the probability of the model correctly predicting the category:

(4) $$FL\left(p_{t}\right)=-\alpha_{t}{\left(1-p_{t}\right)}^{\gamma }log\left(p_{t}\right).$$

Accordingly, the loss function $L_{det}$ for the detection branch can be expressed by Equation (5), where $N_{pos}$ denotes the number of positive samples, which is used for normalization:

(5) $$L_{det}=\frac{1}{N_{pos}}\left(\sum_{i=0}^{N_{pos}}FL\left({\hat{c}}_{i},c_{i}\right)+\sum_{i=0}^{N_{pos}}HB\left({\hat{b}}_{i},b_{i}\right)\right).$$

To address the pose ambiguity caused by symmetric objects, we compute individual losses based on predefined model symmetries and select the symmetry candidate that minimizes the loss as the final result, following Pix2Pose [25]. The set of rotational poses for a symmetric object is denoted as $S_{i}$, where *s* represents a candidate pose in $S_{i}$. To ensure the keypoints branch and the rotation branch are supervised under a unified signal, we introduce the indicator function I(s). This function ensures the symmetry candidate used for I(s) is derived from the keypoints $L_{pts}$. Therefore, the keypoint loss $L_{pts}$, rotation loss $L_{rot}$, and translation loss $L_{tra}$ can be expressed as in Equation (6):

(6) $$\left\{\begin{array}{cc} L_{pts} & =\min_{s\in S_{i}}\frac{1}{N_{pos}}\sum_{i=0}^{N_{pos}}HB\left({\hat{k}}_{i},sk_{i}\right),\\ L_{rot} & =\frac{1}{N_{pos}}\sum_{i=0}^{N_{pos}}HB\left({\hat{R}}_{i},I\left(s\right)R_{i}\right),\\ L_{tra} & =\frac{1}{N_{pos}}\sum_{i=0}^{N_{pos}}HB\left({\hat{T}}_{i},T_{i}\right). \end{array}\right.$$

To maintain the consistency between the predicted keypoints and poses of the network, we additionally introduced the projection loss $L_{pro}$ and the consistency loss $L_{con}$. The formulation can be described as Equation (7), where $K_{3D}$ is projected keypoints at the corners of the object’s bounding boxes:

(7) $$\left\{\begin{array}{cc} L_{pro} & =\frac{1}{N_{pos}}\sum_{i=0}^{N_{pos}}HB\left(k_{i},K_{3D}\hat{p}\right),\\ L_{con} & =\frac{1}{N_{pos}}\sum_{i=0}^{N_{pos}}HB\left({\hat{k}}_{i},K_{3D}\hat{p}\right). \end{array}\right.$$

Thus, for the pose estimation branch of EBFA-6D, the total loss function is shown in Equation (8):

(8) $$L_{pe}=\lambda_{1}L_{rot}+\lambda_{2}L_{tra}+\lambda_{3}L_{pts}+\lambda_{4}L_{pro}+\lambda_{5}L_{con}.$$

For the boundary loss $L_{bdy}$, we observe that the boundary pixels of transparent objects usually occupy a smaller proportion, while the background occupies a larger proportion. To enhance focus on the boundary region and prevent small object features from being overlooked, we use Dice Loss to supervise the boundary branch, expressed as Equation (9):

(9) $$L_{bdy}=1-\frac{2\sum_{x,y}{\hat{B}}_{x,y}B_{x,y}}{\sum_{x,y}{\hat{B}}_{x,y}^{2}+\sum_{x,y}B_{x,y}^{2}}.$$

where x,y represent the position in the pixel coordinate system, while ${\hat{B}}_{x,y}$ and $B_{x,y}$ represent the predicted values and the ground truth, respectively.

### 3.5. Post Processing

Previous methods for decoupling object detection and pose estimation [23,51] have been developed using a two-stage approach. In the first stage, the foreground region is extracted from the input image using an object detector, which integrates non-maximum suppression (NMS) into the object detection framework. In the second stage, the extracted region is fed into a pose estimation network to predict the object’s pose.

In contrast, we propose a single-stage, end-to-end approach that performs pose estimation and object detection in parallel. Since the model makes dense predictions on feature maps at different stages, non-maximum suppression is necessary to eliminate redundant predictions. First, predictions are filtered using a classification score threshold, which was set to 0.75 in our experiments. Next, estimation clustering is performed using the intersection over union (IoU) of the object detection results. The consistency of the model predictions is then used to sort predictions within clusters, with the top *K*, which is set to 10 here, serving as candidates. Finally, the average pose is calculated as the final pose prediction.

## 4. Experiments

This section presents the experimental results of EBFA-6D on the ClearPose dataset. The experiments are conducted using PyTorch and trained on PC equipped with NVIDIA GeForce RTX 4080 GPU (16GB VRAM), 64 GB RAM, and CUDA version 11.8. The Adam optimizer is used to minimize the loss function, with a learning rate of $1\times 10^{-5}$, over a total of 100 training epochs.

### 4.1. Dataset

We use the ClearPose dataset [12], the largest publicly available dataset for transparent object pose estimation, comprising 354,481 real-world RGB-D images of 63 transparent objects captured across 51 distinct scenes. This dataset comprehensively covers the scenarios encountered in daily and includes common vision challenges grouped into six comprehensive challenge sets: New background, Heavy Occlusion, Translucent Cover, Opaque Distractor, Filled Liquid, and Non-planar. For our experiments, we focus on New Background, Heavy Occlusion and Opaque Distractor as test sets to evaluate the performance under diverse and challenging scenarios.

**Figure 5:**
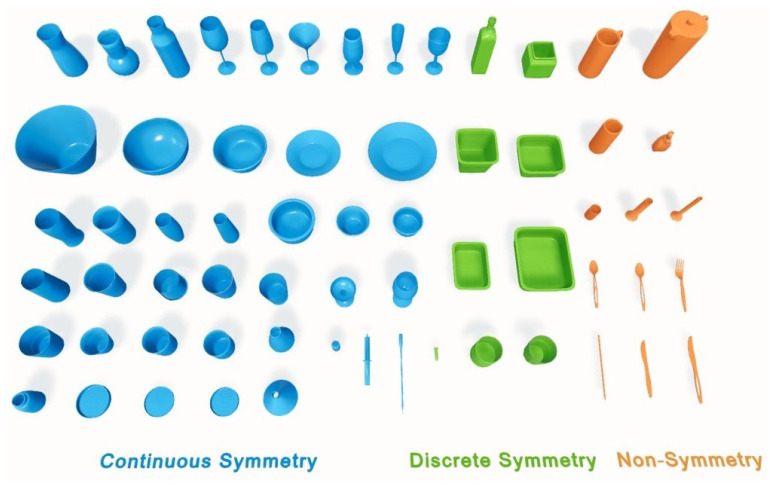
The illustration of the ClearPose dataset. We categorized the 63 instances in the dataset into continuous symmetry, discrete symmetry, and non-symmetry.

ClearPose contains many symmetric objects, such as bowls and wine glasses, which maintain the same appearance when rotated by certain angles around a specific axis. Since our approach is regression-based and supervises the network using one explicit pose, it is necessary to take the symmetry information into account during training to avoid ambiguity. As the authors of ClearPose did not release the symmetry information, we follow the BOP [52] standard to classify and annotate the objects in the ClearPose according to their symmetry properties. As shown in Figure 5, we categorized the objects into three symmetry classes: 13 objects, labeled in orange, do not exhibit symmetry; 9 objects, labeled in green, exhibit discrete symmetry, meaning the object only appears identical when rotated by specific, fixed angles around a particular axis; and 41 objects, labeled in blue, display continuous symmetry, meaning the object looks the same when rotated by any angle around a certain axis. In our experiments, we added labels denoting symmetry categories, axes, and transformation matrix in the annotations.

### 4.2. Evaluation Metrics

To evaluate the performance of the proposed methods in 6D object pose estimation, we use ADD and ADD-S as the pose error functions for non-symmetry objects and symmetry objects, respectively. ADD calculates the average distance between corresponding model points with respect to the predicted pose $\left[\hat{R},\hat{T}\right]$ and ground-truth pose [R,T]. Let *M* represent the 3D model of the object, $V_{M}$ the set of all mesh vertices, and *x* a point on the model *M*. The ADD formula is expressed as Equation (10):

(10) $$e_{ADD}=\frac{1}{|V_{M}|}\sum_{x\in V_{M}}||\left(Rx+T\right)-\left(\hat{R}x+\hat{T}\right){\left|\right|}_{2}$$

ADD-S calculates the average distance to the nearest corresponding point, accounting for rotational symmetry. The formula for ADD-S is shown in Equation (11):

(11) $$e_{ADD-S}=\frac{1}{|V_{M}|}\sum_{x_{1}\in V_{M}}\min_{x_{2}\in V_{M}}\left|\right|\left(Rx_{1}+T\right)-\left(\hat{R}x_{2}+\hat{T}\right){\left|\right|}_{2}$$

We present the results of experiments with ADD(-S) metrics and Accuracy following the protocol in the ClearPose. For Accuracy, if the calculated error is less than 10cm, the prediction is considered correct and Accuracy is the percentage of predictions with ADD<10cm. ADD(-S) is defined as the area-under-curve of accuracy integrated from 0 to 10 cm error scaled by percentage.

### 4.3. Comparison with the State of the Art

We compare our approach with state-of-the-art methods on New Background, Heavy Occlusion, and Opaque Distractor. The methods compare include Xu et al. [39], FFB6D [53], COPE [44], and CDPN [51]. Xu et al. proposed a two-stage method that first addresses depth errors through surface normals and applying RANSAC-based plane estimation, followed by utilizing an extended point-cloud representation to achieve precise and efficient pose estimation. FFB6D employs a bidirectional fusion strategy to process RGB-D images, effectively integrating appearance and geometric information in a unified encoder–decoder architecture. CDPN adopts a two-stage approach which first utilizes a detector to locate objects, then densely regressing 3D objects coordinates, followed by the RANSAC-PnP to solve object poses. COPE is an end-to-end network that directly predict the 6D pose from RGB images. COPE and CDPN are both trained on the ClearPose training set, and evaluated on RTX4080. We utilize YOLOv5 [54] as the detector for CDPN, noting that detection time is not included in the reported inference time for CDPN. The results for Xu et al. and FFB6D are obtained from the ClearPose [12], where both methods are trained and tested on raw depth data.

Table 1 illustrates the superior accuracy and speed of EBFA-6D compared to other methods. Compared to CDPN, EBFA-6D not only achieves higher accuracy and ADD(-S) scores, but also performs inference nearly 18 times faster. Compared to COPE, EBFA-6D achieves approximately 10% higher accuracy and ADD(-S) across all test sets. Despite having nearly two million more trainable parameters than COPE, EBFA-6D is only 0.017 s slower during inference. In comparison to Xu et al. and FFB6D, EBFA-6D demonstrates a clear accuracy advantage on New Background and Heavy Occlusion test sets. Furthermore, we observe a performance disparity in the Opaque Distractor set, which is captured in significantly different environmental conditions. While FFB6D struggles in these scenarios due to interference caused by opaque objects affecting the depth of transparent objects, RGB methods, including ours, experience a performance drop caused by shift in color space distributions. In contrast, Xu et al.’s two-stage approach demonstrates robustness by leveraging depth completion.

### 4.4. Ablation Experiments

We evaluated the effectiveness of the proposed modules using New Background as the test set on ablation experiments. As BFAM consists of TBPM and BFM, we isolate the two submodules and individually evaluate their impact model’s performance. We reimplemented the baseline following COPE using PyTorch 1.13, achieving better metrics compared to COPE’s original experiments.

As shown in Table 2, our lightweight boundary predictor, TBPM, introduces only a slight computational overhead. Incorporating boundary prediction as an additional task benefits the pose estimation accuracy. When the BFM is integrated into our framework without boundary prediction, its channel attention mechanism also boost the model’s performance. Combining both submodules (TBPM and BFM) in the BFAM results in significant performance improvements, achieving 7.75% higher recall and 6.56% higher accuracy compared to the baseline, albeit with an increase in inference time. BFFM enhances the model with 3.33% higher recall, 2.91% higher accuracy, and a minimal impact on inference speed. When combined, they achieve 12.72% higher recall, 10.27% higher accuracy and 7.71% higher ADD(-S), with only a 0.0185 s increase in inference time compared to the baseline. Through experiments involving various combinations of these components, we demonstrate that their integration is crucial to the effectiveness of our method.

**Figure 6:**
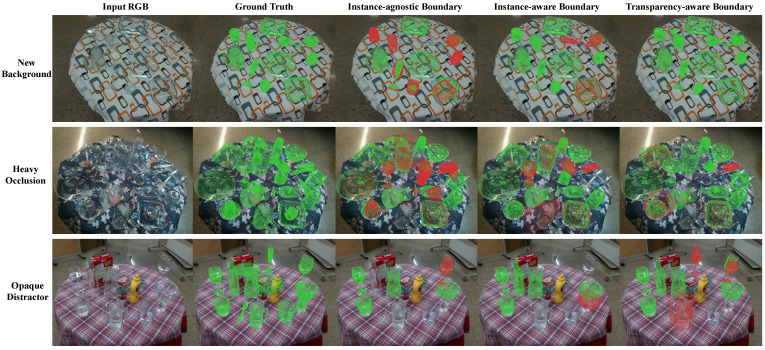
Comparative results of the three pixel-level supervision approaches. The experiment is tested on three different test sets of the ClearPose. The green represent correct pose estimations, while the red represent incorrect pose estimations.

We also conducted comparative experiments with the three pixel-level supervision approaches discussed in Section 3.2.1 to evaluate the effectiveness of the transparency-aware boundary in TBPM. As shown in Figure 6, the instance-agnostic boundary approach performed the worst. This was primarily due to the blurring of boundaries between instances, which hindered the model’s ability to distinguish individual objects. The instance-aware boundary method improved performance by leveraging boundary pixels to help the model focus on transparent object boundaries. However, it suffered from missed or misclassified objects, particularly for occluded instances with incomplete boundaries. In contrast, the transparency-aware boundary approach achieved the best results, demonstrating that per-object constraints combined with boundary information effectively reduced ambiguity.

## 5. Discussion

**Figure 7:**
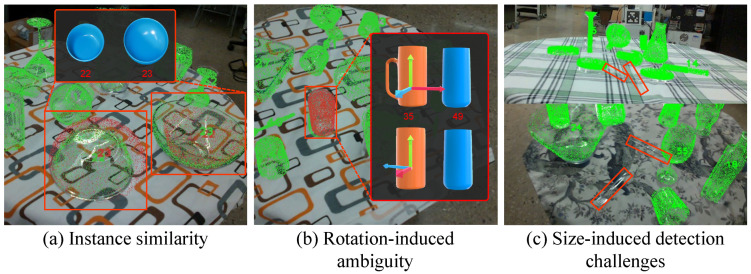
Examples of failure cases for EBFA-6D on the ClearPose dataset. (**a**) Instance similarity: Instances 22 and 23 show slight curvature differences at the bottom, which the model struggles to distinguish. (**b**) Rotation-induced ambiguity: Instance 35 resembles instance 49 after a 90° rotation, leading to confusion. (**c**) Size-induced detection challenges: Small and slender objects such as knives, spoon and stirring sticks are frequently under-detected or misclassified.

EBFA-6D has successfully achieved a balance between speed and accuracy compared to previous methods, but still underperforms on the ClearPose dataset. We observe that the model tends to misclassify instances such as 22 and 23, as well as 35 and 49. By comparing the original images of these instances with the model’s predictions, we identify three causes for errors, as shown in Figure 7.

1. Instance similarity. Due to the lack of depth information and high-resolution details, object instances with similar appearances but different sizes become difficult to distinguish.
2. Rotation-induced ambiguity. Certain object instances can be easily identified when viewed from specific angles. However, when critical visual cues are obscured, their distinction becomes ambiguous.
3. Size-induced detection challenges. Small or slender objects have limited pixel coverage in the input image, limiting the available information for detection. This leads to frequent under-detection or misclassification by the model.

In summary, incorrect detection and classification of transparent objects are the primary reasons for the model’s reduced accuracy. The ability to effectively extract and use small features in the input image is key to addressing this issue. Thus, future improvements should focus on enhancing the model’s capacity to detect and utilize fine details.

In the future, leveraging synthetic data offers a promising avenue for transparent object pose estimation. Synthetic environments can be easily constructed, providing greater diversity and control over object scenes, enabling models to learn generalized features. Furthermore, since transparent objects within the same category often share similar appearances and are difficult to distinguish at the instance level, adopting category-level pose estimation can improve generalization to unseen objects within known categories, significantly enhancing practicality for diverse applications.

## 6. Conclusions

In this work, we present an end-to-end framework EBFA-6D to address the challenges of transparent object pose estimation. The key idea is to leverage the consistent boundary information to enhance representation learning for transparent object perception. Additionally, we integrate a bottom-up feature fusion module that preserves low-level features while enriching high-level semantic features, which further boosts the performance. Our model is capable of estimating multiple object poses with a single forward pass, making it well-suited for real-time applications. In future work, we aim to further enhance the model’s generalization capabilities by increasing scene diversity through synthetic data, as well as explore category-level approaches to extend the applicability of 6D pose estimation.

## Figures and Tables

**Table 1:** Comparison with different pose estimation methods on the ClearPose dataset using accuracy, ADD(-S), and average time.

Method	Input	New Background		Heavy Occlusion		Opaque Distractor		Time (s)
		Accuracy	ADD (-S)	Accuracy	ADD (-S)	Accuracy	ADD (-S)	
Xu et al. [39]	RGB-D	50.96	45.23	24.19	22.95	**42.63**	**39.04**	-
FFB6D [53]	RGB-D	44.26	43.45	14.72	17.87	0.46	0.76	-
CDPN [51]	RGB	58.57	52.88	38.20	36.91	26.48	23.25	1.4660
COPE [44]	RGB	51.48	49.75	35.07	32.69	20.73	17.54	**0.0623**
Ours	RGB	**62.43**	**58.03**	**44.91**	**40.53**	31.62	26.89	0.0793

**Table 2:** Ablation study of EBFA-6D. In the TBPM experiment, the BFM is removed and the boundary serves solely as an additional supervision signal. In the BFM experiment, boundary prediction is disabled and TBPM is modified such that $F_{i}$ is directly passed through a 3 × 3 convolutional layer followed by the channel attention.

Methods	Recall	Accuracy	ADD (-S)	Time (s)
Baseline	31.47	52.16	50.32	0.0608
TBPM	33.54	53.91	51.76	0.0632
BFM	35.23	56.18	53.94	0.0653
BFAM	39.22	58.72	55.80	0.0696
BFFM	34.80	55.07	52.11	0.0647
BFAM + BFFM	44.19	62.43	58.03	0.0793

## Data Availability

Publicly available datasets were analyzed in this study. This data can be found here: https://github.com/opipari/ClearPose, accessed on 3 October 2024.

## Abbreviations

The following abbreviations are used in this manuscript:

BFAM	Boundary Feature Augmented Mechanism
TBPM	Transparency-Aware Boundary Prediction Module
BFM	Boundary Focus Module
BFFM	Bottom-up Feature Fusion Module
ADD	Average Distance of Model Points
ADD-S	Average Distance of Model Points—Symmetric objects
ADD(-S)	Average Distance of Model Points—with Symmetry Handling
